# Tumor and reproductive traits are linked by RNA metabolism genes in the mouse ovary: a transcriptome-phenotype association analysis

**DOI:** 10.1186/1471-2164-11-S5-S1

**Published:** 2010-12-22

**Authors:** Ulises Urzúa, Garrison A Owens, Gen-Mu Zhang, James M Cherry, John J Sharp, David J Munroe

**Affiliations:** 1 Laboratorio de Genómica Aplicada, ICBM, Universidad de Chile, Independencia 1027, Santiago, Chile; 2 Laboratory of Molecular Technology, SAIC-NCI Frederick, 915 Tollhouse Avenue, Suite 211, Frederick, MD 21701, USA; 3 Laboratory Animal Sciences Program, SAIC-NCI Frederick, Frederick, MD 21702, USA; 4 Center for Comparative Medicine, Baylor College of Medicine, One Baylor Plaza, Houston, TX 77030, USA

## Abstract

**Background:**

The link between reproductive life history and incidence of ovarian tumors is well known. Periods of reduced ovulations may confer protection against ovarian cancer. Using phenotypic data available for mouse, a possible association between the ovarian transcriptome, reproductive records and spontaneous ovarian tumor rates was investigated in four mouse inbred strains. NIA15k-DNA microarrays were employed to obtain expression profiles of BalbC, C57BL6, FVB and SWR adult ovaries.

**Results:**

Linear regression analysis with multiple-test control (adjusted *p* ≤ 0.05) resulted in ovarian tumor frequency (OTF) and number of litters (NL) as the top-correlated among five tested phenotypes. Moreover, nearly one-hundred genes were coincident between these two traits and were decomposed in 76 OTF(–) NL(+) and 20 OTF(+) NL(–) genes, where the plus/minus signs indicate the direction of correlation. Enriched functional categories were RNA-binding/mRNA-processing and protein folding in the OTF(–) NL(+) and the OTF(+) NL(–) subsets, respectively. In contrast, no associations were detected between OTF and litter size (LS), the latter a measure of ovulation events in a single estrous cycle.

**Conclusion:**

Literature text-mining pointed to post-transcriptional control of ovarian processes including oocyte maturation, folliculogenesis and angiogenesis as possible causal relationships of observed tumor and reproductive phenotypes. We speculate that repetitive cycling instead of repetitive ovulations represent the actual link between ovarian tumorigenesis and reproductive records.

## Background

Epidemiological evidence indicates that multiparity and breastfeeding as well as endocrine disrupting agents -used in oral contraception, hormone replacement therapy and infertility treatment- modulate the risk of ovarian cancer [1]. Repetitive lifetime ovulations would induce a persistent wound repair process of the ovarian surface epithelium cells leading to pre-neoplastic alterations [2]. In addition, oral contraceptives and pregnancy reduce levels of circulating gonadotropins whereas fertility drugs induce follicle-stimulating hormone (FSH) production. Gonadotropins also increase with reproductive ageing, and have been implicated in ovarian cancer etiology since this malignancy predominantly occurs in menopausal women [3].

The laboratory mouse has been increasingly used to model several aspects of ovarian cancer [4]. Indeed, reproductive biology of mouse resembles human reproduction in many aspects. Analogous to menstrual cycles in women, female mice undergo estrous cycles that last 4-5 days and consist of four successive phases. Proestrus and estrous phases together constitute the follicular phase while metestrus and diestrus phases together represent the luteal phase [5]. Similar to humans, the length of estrous cycles increases while the monthly cycle frequency decreases in ageing mice [6]. The number of ovulations roughly reaches 500 during the reproductive life of women while in mice this number can be achieved earlier than middle age due to multiple ovulations in a single cycle [7] as judged by the litter size observed in mice [5]. Cysts, invaginations and cell layering are also common observations in the mouse and human ovaries [7].

Mouse inbred strains display measurable traits that are described as continuous phenotypes in the Mouse Phenome [8] and the Mouse Tumor Biology [9] databases. The natural variability observed in mice strains offers the opportunity to study disease susceptibility in a genetically defined background. In simple terms, phenotypic variability could be due to the interplay of gene transcripts and/or proteins expressed at different relative abundances across individuals in a tissue or cell type implicated in a phenotype. Thus, if a correlation exists between a continuous phenotype and gene expression, a measure of each gene’s contribution to the observed phenotype can be inferred. DNA microarrays are suited to measure transcript levels for hundreds or thousands of genes simultaneously, and thus such contribution can be addressed in a wide-genome format. A number of statistical approaches have been recently formulated to correlate DNA microarray data with phenotypic covariates [10].

In an attempt to gain novel information linking reproductive parameters with ovarian tumorigenesis we describe here a correlation analysis between spontaneous ovarian tumors, reproductive phenotypes and gene expression profiles obtained with NIA15k-DNA microarrays from ovaries of four mouse inbred strains. Using a linear regression approach with control of multiple testing, “ovarian tumor frequency” (OTF) and “number of litters” (NL) were the top-correlated of 5 analyzed phenotypes. About one hundred genes were coincident between OTF and NL. The enriched biological functions in this overlapped sub-set were “RNA-binding/mRNA-processing” and “protein folding”. The relevant information concerning the significant genes was mined and the relationship between ovarian function and ovarian tumorigenesis at the molecular level is discussed.

## Results

### Consistency of microarray data and Q-PCR assays

**Figure 1:**
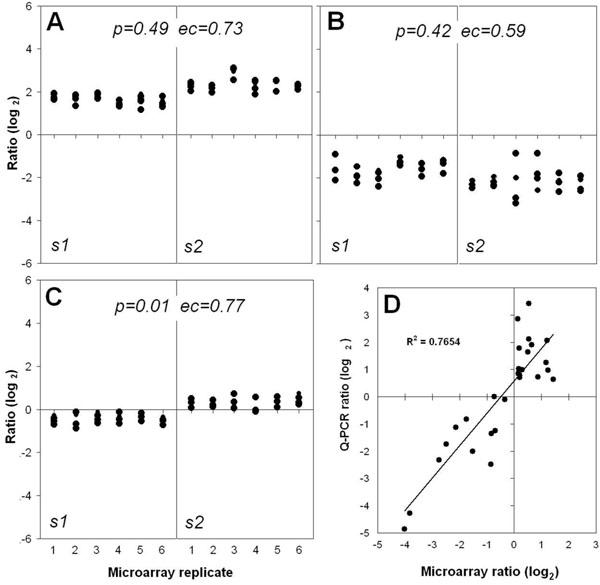
**Consistency of microarray results and Q-PCR confirmation of selected genes** Normalized microarray data of 5 cDNA clones of *Tsc22* (A), 4 clones of *Col3a1* (B) and 4 clones of *Fubp1* (C) were compared between samples s1 and s2 (corresponding to 2 of the 4 mouse ovarian samples assayed) across 6 microarray replicates each. Adjusted *p*-values and expression change (ec), defined as the difference between log_2_ averages are shown. In D, the coordinates of scatter plot depicts the average of normalized microarray ratios (5-6 replicates) and the average of duplicate Q-PCR assays for the genes *Spp1*, *Txnrd1*, *Anxa5*, *Fn1*, *C1s*, *Ctsl*, and *Mt1* in each of the 4 mouse strains. The internal control transcript was the 18S rRNA. Q-PCR data was converted to log_2_ scale ratios as described in Methods. The squared correlation coefficient (R^2^) shown corresponds to the overall gene subset in the 4 strains (i.e. 28 data pairs). Raw Q-PCR results and individual microarray ratios are detailed in Additional file1, table 1.

The robustness and reliability of the mouse NIA-15K cDNA microarray platform has been demonstrated in our previous work [11,12] and by others [13-15]. Experimental design employed a common reference RNA and replicate dye-swap. Raw data was subjected to print-tip loess normalization, a numerical correction based on local deviations across the microarray surface aimed to counteract the inherent noise of these devices [16]. Further adjustment consisted of inter-slide scale normalization, after which statistical comparisons were performed. The complete normalized dataset for 14,586 cDNA clones in 23 microarrays corresponding to 4 samples (i.e. 6 replicates for 3 samples and 5 replicates for 1 sample) is available as a Supplementary spreadsheet (Urzua_complete_dataset.xls) and has been deposited to the GEO database (http://www.ncbi.nlm.nih.gov/geo/) with the accession code GSE18045. One of the 24 hybridization experiments was discarded because not meeting with minimal image quality parameters. Figure 1, panels A through C, shows microarray data comparison for expression of genes *Tsc22*, *Col3a1* and *Fubp1* in 2 strains assayed each in 6 microarray replicates. These genes were selected because they were present as multiple cDNA clones in the NIA-15K collection, so that intra-slide consistency could also be evaluated. The expression change (ec), defined as the difference between log_2_ ratio averages of all clones in the 6x2 replicate arrays, was similar among the 3 genes. However, the adjusted *p* value was significant for the *Fubp1* gene only. Additionally, quantitative-PCR (Q-PCR) confirmation assays were performed for seven genes assessed in a previous mouse ovarian study, and for which primer pairs were available. Three of these genes showed statistical significance after an ANOVA test across the 4 mouse strains (see next section). The C_T_ values for test and reference samples were corrected with the 18S-rRNA as internal control transcript and then converted to log_2_-based ratios to compare with microarray results. As shown in Figure 1D, the squared correlation between Q-PCR and microarray platforms was R^2^ = 0.749, a value over the range observed in a recent large-scale study aimed to validate microarray data using Q-PCR [17]. Microarray data roughly ranged from -4.0 to +1.5 while the correspondent Q-PCR results ranged from -4.9 to +3.4. Except for one data pair, out of 28 comparisons, the ratio direction (up- or down-regulation) more than the absolute value, was consistent between both methodologies. Additional file 1, Table 1 (Urzua_Suppl_Results) shows detailed microarray log_2_ ratio values and C_T_ values for the seven genes in all test and reference samples.

### Tumor and reproductive parameters correlated with ovarian gene expression

**Figure 2:**
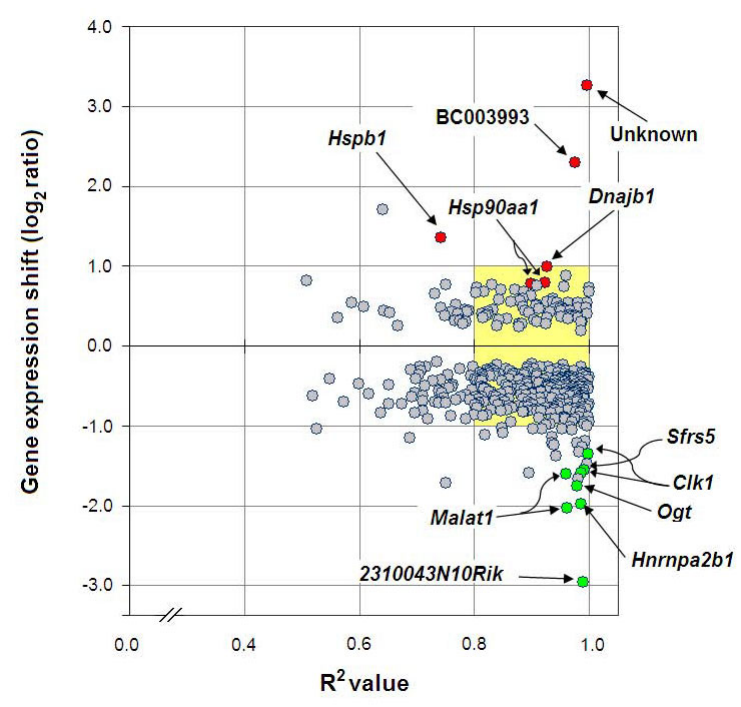
**Strength of OTF correlation as a function of ovarian gene expression shift** The squared correlation coefficient (R^2^) for the 425 clones positively and negatively correlated (adjusted *p*<0.05) with ovarian tumor frequency (OTF) were plotted against their respective gene expression shifts. The shift is defined as the resultant of the subtracted gene expression log_2_ ratios between extreme values (see Table 1 and Additional file 1, 2, 3 Results file).

A preliminary ANOVA analysis resulted in 628 cDNA clones (4.27 % of the whole dataset) with statistically significant differences (adjusted *p*<0.05) between 2 of any of the four mouse ovarian tissue profiles (data not shown). These results simply indicate that transcriptional differences indeed occur among ovarian tissue across strains. Thus, in order to add physiological meaning to this observation, the whole microarray dataset was subjected to a linear regression analysis with each of the continuous covariates shown in Table 1. A summary of regression results is shown in Table 2. Using an adjusted *p* value <0.05 as cut-off, a total of 590 clones that represented 401 known genes, 93 unknown genes, 45 transcribed locus and 9 expressed sequences were significantly correlated to 4 of the 5 traits analyzed. The overlap between the regression analysis and the ANOVA test was 386 clones. The phenotype productive matings (PM) did not result in any significantly correlated gene. The gene ontology (GO) profile of the 401 known genes included “regulation of transcription” (67 genes; *p*= 4.4e-7), “RNA binding” (44 genes; *p*= 4.3e-16) and “RNA metabolism” (26 genes; *p*= 1.0e-7) with the highest statistical significance in a hypergeometric test. Notably, even though GO terms are inherently redundant, the combined 3 functional groups corresponded to 26% (105) of known significant genes. The overall functional profile of the 401 genes list is detailed in Additional file 1, table 3. Ovarian tumor frequency (OTF) was the top correlated trait followed by number of litters (NL). The relationship between the squared correlation coefficient (R^2^) for each of the 425 OTF correlated clones and its corresponding gene-expression shift is depicted in Figure 2. Negative and positive correlation was observed for 328 and 97 clones, respectively. Notably, a large fraction of strongly correlated genes (R^2^ = 0.8 to 1.0) showed gene-expression shifts under 1 unit in the log_2_ ratio scale (mean +0.53 and -0.58 in the yellow highlighted quadrants) which equals to less than 2-fold up- or down-regulation in the linear scale across the 4 strains. Selected clones either with high significance, large expression shift or both are tagged with red and green dots in Figure 2. OTF positively correlated genes included the unknown function H3055D10 clone, the BC003993 clone coding for the KIAA1604 protein presumably implicated in nuclear mRNA splicing [18], the heat shock proteins *Hspb1*, *Hsp90aa1* and *Dnajb1* involved in protein folding and cellular stress [19], and the gene *Star* (steroidogenic acute regulatory protein), which mediates mitochondrial cholesterol transport for its conversion to pregnenolone [20]. On the other hand, genes negatively correlated with OTF included *Ogt*, coding for a N-acetylglucosamine transferase enzyme activity implicated in heat-stress response [21], the mRNA splicing genes *Hnrnpa2b1*, a possible early detection marker of lung cancer [22], and *Sfrs5* which is overexpressed in breast tumors [23]. Additional OTF(-) genes included 2310043N10Rik corresponding to a virus-inducible non-coding RNA (VINC) expressed in brain and several adult non-neuronal mouse tissues [24], the two clones of *Malat1*, a long, non-coding metastasis-associated lung cancer transcript up-regulated various tumors including ovarian cancer [25], and *Clk1* (CDC-like kinase 1), involved in nuclear phosphorylation of serine/arginine-rich proteins in the spliceosomal complex [26].

**Table 1:** Ovarian tumor and reproductive phenotypes in selected mouse strains

Strain	Tumor frequency**^a^**	Litter size**^b^**	Number of litters	Productive matings (%)	Relative fecundity
BALB/c	3.80	4.9	3.6	55.6	9.80
C57BL/6	1.60	6.6	3.8	87.4	21.9
FVB**^c^**	7.00	9.5	4.8	90.0	41.0
SWR	57.0	7.5	2.3	58.3	10.1

**^a^** Refers to spontaneously arisen tumors in inbred mice. Data corresponds to the “highest reported tumor frequency” in all literature records collected in the Mouse Tumor Biology Database [9], (http://tumor.informatics.jax.org/mtbwi/dynamicGrid.do;jessession=89370725979E9B939D3DD40AB4961BA5) for each strain/organ combination where organ=ovary.

**^b^** Litter size, number of litters, and productive matings were taken from the Mouse Phenome Database [8] (http://www.jax.org/phenome). Data acquisition, curation and handling are described at  http://phenome.jax.org/db/q?rtn=docs/aboutmpd. The parameter “relative fecundity” is derived form the other 3 reproductive parameters [5].

**^c^** Data of FVB strain was taken from Silver’s Mouse Genetics textbook [5].

**Table 2:** Summary of correlation results between ovarian gene expression and phenotypes

Trait	Correlated clones**^a^**	Trait interactions**^b^**				Direction and strength of correlation (R value range)**^c^**		Gene expression shift (δ log_2_ value range)**^d^**	

		OTF	NL	LS	RF	Positive	Negative	Positive	Negative
Ovarian tumor frequency (OTF)	425 (280)	-	145	0	0	97 (1.00-0.51)	328 (1.00-0.52)	3.26 - 0.19	2.96 - 0.21
						
Number of litters (NL)	234 (82)	145	-	0	7	161 (1.00-0.73)	72 (0.99-0.67)	2.94 - 0.32	3.00 - 0.24
						
Litter size (LS)	73 (66)	0	0	-	7	53 (0.99-0.78)	20 (0.99-0.62)	2.50 - 0.32	2.18 - 0.41
						
Relative fecundity (RF)	17 (3)	0	7	7	-	12 (0.98-0.78)	5 (0.98-0.90)	1.50 - 0.29	0.85 - 0.51

**^a^** Number of clones resulting from a regression analysis with false discovery rate (FDR) control performed with the multiple test tool Pomelo (accessible at http://pomelo2.bioinfo.cnio.es/). For each trait, genes were filtered with an adjusted p<0.05 resulting in a total of 590 unique arrayed clones showing statistically significant correlation. Under this criterion, the trait “productive matings” did not show correlation. The number of clones exclusively correlated with the indicated trait is shown between parentheses.

**^b^** Interactions correspond to the number of clones correlated with two or more traits as obtained with Boolean comparisons.

**^c^** Pearson correlation coefficients (R) were calculated using gene expression log_2_ ratios as independent variable and each of phenotypic trait (see Table 1) as dependent variables. The observed range of R values for all correlated genes is shown between parentheses.

**^d^** Gene expression ratios (log_2_) for the extreme trait values were subtracted (δ) and then ranked. The highest and the lowest ratio difference are shown between parentheses.

### Functional analysis of genes associated both to OTF and NL

**Figure 3:**
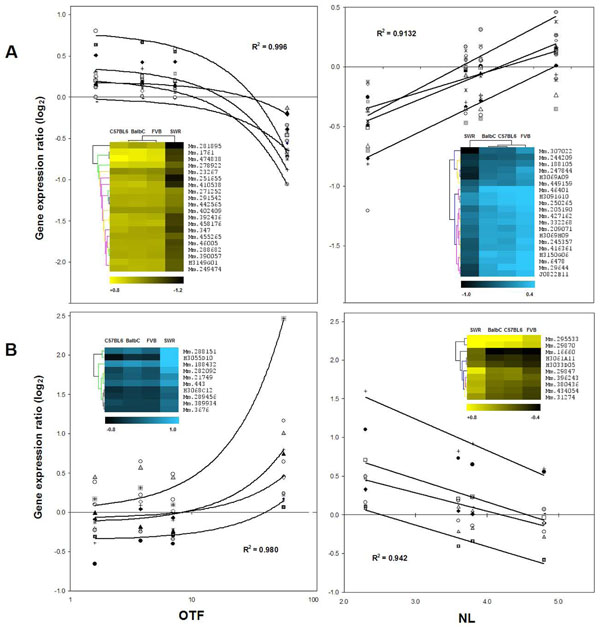
**Ovarian gene expression correlated to OTF and NL** The gene expression ratios of the top-20 OTF(-), NL(+) best correlated clones (A) and the top-10 OTF(+), NL(-) best correlated clones (B) are plotted against their respective phenotypic variables. The average of squared correlation coefficients and four representative tendency lines are shown in each plot. Insets show hierarchical clusters for genes (vertical trees) and samples (horizontal trees). Yellow clusters represent negative correlation and blue clusters represent positive correlation. Color scales and Unigene IDs are shown at the bottom and right side of each cluster, respectively. Unknown clones appear with the NIA-15K clone code.

Table 2 also shows the number of genes correlated with more than one trait. The highest overlap was 145 clones associated both with OTF and NL. No overlap was observed between OTF and litter size (LS), or between OTF and relative fecundity (RF). The latter parameter -numerically derived from NL, LS and PM [5] - resulted in 17 correlated genes, 14 of them also correlated to NL and LS, 7 genes each trait. Interestingly, LS showed the highest percentage (90%) of exclusively correlated genes. Figure 3 shows scatter plots for a combined total of 40 OTF and 20 NL correlated transcripts. Genes with the top correlation coefficients (R) and equivalent distribution of positive and negative correlation are shown. R values of negatively correlated clones ranged -0.999 to -0.798, and +0.998 to +0.875 for the positively correlated ones. Similarly, R values for NL correlated genes ranged -0.986 to -0.845 and +0.999 to +0.756 [Additional file 1, 2, 3 “OTF&NL_correlated_transcripts.xls”]. Following exclusion of unknown clones and merging of repeats, the OTF/NL-145 clones list was reduced to 117 single Unigene IDs including 21 transcribed loci of known chromosomal location but unassigned biological function. Thus, the final list actually consisted of 96 unique gene identities and was further decomposed in 2 sub-lists based on direction of correlation: 76 OTF(-) NL(+) genes and 20 OTF(+) NL(-) genes, where the minus sign indicates negative correlation and the plus sign indicates positive correlation. The genes in enriched functional categories (56 out of the 96) are shown in Additional file 4. Overall, the predominant functions were related to RNA-metabolism (i. e. RNA binding, mRNA processing, regulation of transcription, zinc ion binding) and protein folding and degradation. Eleven of the 56 genes have not been linked to a defined cellular or physiological process since they comprise recently identified genes for which GO terms were inferred from electronic annotation. Importantly, 15 genes are linked to normal or pathological ovarian processes, including *Cited1* and *Ece1* directly implicated in reproductive functions. Further links to reproduction involve the genes *Aplp2*, *Dnaja1*, *Htt*, *Rps6kb1* and *Spin1* which were part of the overall correlated 590 clones subset (see Discussion). In addition, as shown in Additional file 4, five genes have been described in normal or altered hematopoiesis, 5 genes are associated to neurological disorders, 3 genes are muscle-related and 3 genes are linked to non-ovarian endocrine function.

## Discussion

Complex phenotypes are the outcome of many genes interacting with each other and with endogenous or exogenous factors. Mouse strains displaying phenotype variability allow interrogation on their molecular basis in a particular tissue or condition. In this report, the ovarian expression of roughly 400 genes (corresponding to 590 transcripts in Table 2) was significantly correlated to 4 of 5 mouse tumor and reproductive phenotypes assessed with a linear regression model. The predominant gene ontology (GO) terms were “regulation of transcription”, “RNA binding” and “RNA metabolism” accounting for 105 of all correlated genes. A minor, but significant group was “ubiquitin cycle” with 14 genes. Links to reproductive processes are described for *Aplp2*, *Chuk*, *Dnaja1*, *Htt*, *Pten*, *Rps6kb1*, *Sf1*, *Spin1* and *Tnc* in the GO directory. *Rps6kb1* is involved in proliferation of granulosa cells in response to FSH [27]. *Rps6kb1* and *Chuk*, in addition to *Nfkb1*, *Map3k10*, *Flna*, *Kras*, *Rap1*a, and *Hspb1* belong to the MAP kinase signaling pathway which has been implicated in mammalian oocyte maturation and fertilization [28]. The correlation of *Kras* (K-Ras 2), commonly mutated in various human tumors, with litter size (LS) can be supported by its involvement in granulosa cell differentiation and ovulation [29].

In a study on null *Foxo3* mice, a mutant displaying early ovarian hyperplasia due to synchronous primordial follicle activation, 6 genes (*Spin1*, *Slc45a3*, *Rspo2*, *Star*, *Trim71* and *Gm196*) present in our 400-genes list were postulated as fertility factors [30]. *Star* (steroidogenic acute regulatory) protein was positively correlated to OTF (see Figure 2). *Star* transports cholesterol into the mitochodria, a key process in steroid-hormone synthesis in all major steroidogenic tissues [20]. Additional genes related to steroid metabolism and present in the 400-list included *Hmgcr* (3-hydroxy-3-methylglutaryl-CoA reductase), the major regulatory step in cholesterol synthesis; *Idi1* (isopentenyl-diphosphate delta isomerase) involved in conversion of mevalonate into activated isoprene units, and *Lss* (lanosterol synthase) that catalyzes the cyclization of squalene-2,3-epoxide to lanosterol. A recent work has implicated metabolic products of lanosterol in primordial folliculogenesis by regulation of oocyte meiosis and apoptosis [31]. Other indirectly steroid-related gene was *Mbtps2*, a membrane-embedded zinc metalloprotease which activates signaling proteins involved in transcription induced by steroids [32].

A large portion of the 590 list (24.6%, i.e. 145 clones) was found to be associated both to spontaneous ovarian tumor frequency (OTF) and number of litters (NL) as shown in Table 2. A link between OTF and NL agrees with an increased risk of ovarian tumorigenesis due to successive menstrual cycles in women. In contrast, conditions that interrupt cycles block ovulations and thus reduce risk [2]. Accordingly, a mouse strain displaying high NL has been subjected to a longer period without cycling than compared with a low NL strain. Successive pregnancies and lactation may be responsible of this effect. We detected a set of 76 mouse genes that were positively correlated to NL, i.e. elevated expression levels were observed in strains showing high NL. Thus, since a concomitant negative correlation was observed with ovarian tumor frequency (OTF), over-expression of these 76 genes set could be considered “protective”. By analogy, the 20 genes that were negatively correlated to NL, i.e. down-regulated in high NL mice, may have a role as “susceptibility” genes since they showed a parallel positive correlation with OTF. Importantly, high litter sizes (LS) involve multiple simultaneous ovulations but no association was detected between OTF and LS in our data. It may be hypothesized that the damage caused to the epithelial surface during a multiple-ovulation event can be repaired during subsequent pregnancy and lactation, a period without cycling and ovulations.

Additional file 4 shows that 11 of the 76 OTF–/NL+ genes are implicated either in normal or pathological ovarian processes. RNA binding was the predominant GO term in the 76-genes list reaching 22 genes. RNA binding proteins have been implicated in mammalian germ cell development [33]. The genes *Cpsf6*, *Ddx17*, *Fubp1*, *Hnrpa2b1*, *Rbm25*, *Rbm39*, *Sfrs2* and *Sfrs6* share GO terms (RNA binding, mRNA processing) and a relationship with normal ovarian function or ovarian-related disease. Some genes not related to RNA metabolism but linked to ovarian function were correlated to OTF–/NL+. These include *Ece1*, expressed in steroidogenic and follicular endothelial ovarian cells in a parallel fashion to corpus luteum maturation [34], and the genes *Arnt* and *Nfat5* that regulate the activity of vascular endothelial growth factor (VEGF), an important angiogenesis modulator in normal and pathological conditions including ovarian malignancies [35]. *Arnt* (alias HIF-beta) binds the hypoxia inducible factor-1 alpha (HIF-1alpha) to form a heterodimer that recognizes the VEGF promoter [36]. *Arnt* can alternatively bind the aryl hydrocarbon receptor (AHR) forming an AHR/ARNT complex which controls FSH and LH concentrations in response to AHR ligands [37]. Furthermore, down-regulation of *Nfat5* (nuclear factor of activated T-cells 5) parallels a decrease in VEGF’s receptor VEGFR1 and an increase in VEGFR2 in hemangioma endothelial cells [38].

Additional regulators of VEGF’s function in the OTF–/NL+ list were the genes *Sfrs6* and *Rbm39*. The splicing factor *Sfrs6* (alias SRp55) upregulates the anti-angiogenic VEGF isoform, an alternative splicing product involving the 8^th^ exon of VEGF’s pre-mRNA [39]. Since *Sfrs6* displayed an OTF–/NL+ correlation pattern, this is suggestive of an anti-angiogenic condition associated to multiparity and low ovarian tumor incidence. VEGF levels itself were neither differentially expressed nor correlated to any phenotype in this study, but the CDC-like kinase 1 (*Clk1*), which mediates *Sfrs6* activity on anti-angiogenic VEGF levels [39], was also part of the OTF–/NL+ list (see Additional file 3 “OTF&NL_correlated_transcripts.xls”). In addition, *Rbm39* (alias CAPERalpha) is able to alter the VEGF-121/VEGF-189 ratio in breast cancer cells [40]. *Rbm39* was originally described as an ERα/β transcriptional coactivator [41]. Analogous role has been reported for the DEAD-box RNA helicases *Ddx17* (alias p72) and *Ddx5* (alias p68). [42]. *Ddx17* was in a OTF–/NL+ fashion while *Ddx5* plus *Ddx26b*, *Ddx3x* and *Ddx42* were present in the overall 400-genes list.

Regarding the OTF+/NL– correlated genes, the predominant GO term was “protein folding” including *Dnajb1*, *Hsp90aa1* and *P4hb* (see Additional file 4 and Figure 2). *Dnajb1* is a member of the Hsp40 co-chaperone protein family of which its *Drosophyla* homolog participates in oogenesis [43]. Related Hsp-40 genes *Dnaja1*, *Dnajb6*, and *Dnajc7* were present in the overall 400-genes list. *Hsp90aa1* expression is up-regulated in ovarian endometriosis [44] while *P4hb* is involved in post-translational modifications of procollagen synthesis [45]. Other transcripts with OTF+/NL– correlation were *Spin1* (Spindlin 1), a protein that associates to CPEB, a RNA-binding protein implicated in polyadenylation during meiotic progression in oocytes [46], and the transcription factor *Cited1*, reported as a FSH regulated gene in human granulosa cells [47].

All the five phenotypes studied are complex and many causal effects are certainly involved. It is quite possible that a large fraction of correlated transcripts may be simply bystanders but not lie behind the measured phenotype. Of the 56 genes listed in Additional file 4, seventeen are classified under the GO term “regulation of transcription”. These may be considered “master genes”, i.e. encoding for protein products that somehow interact with DNA regulatory sequences or transcriptional multiprotein complexes thus modulating the transcriptional activity of downstream genes. Among the OTF–/NL+ correlated genes with roles in regulation of transcription, we identified *Fubp1*, *Rbm39* and *Arnt* which are directly linked to ovarian biology or disease (see Additional file 4). *Fubp1* encodes a ssDNA binding protein that activates the “far upstream element” of c-myc thus stimulating its transcription. Interestingly, promoter regions of the OTF–/NL+ genes *Ccnl1*, *Clk4*, *Coq10a*, *Ddx17*, *Ict1*, *Zc3h11a* and *Zc3h7a* were found to contain binding sites for *Arnt* (data not shown). In addition, the genes *Hnrpdl*, *Sltm*, *Tardbp*, *Ccnl1*, *Ccnl2*, *Dmtf1*, *Mll3*, *Mycbp2*, *Nfat5*, *Suv420h2*, and *1810007M14* display diverse roles in cancer and developmental processes (see Additional file 4 for References). Special mention deserve the c-myc binding protein *Mycbp2*, the gene *Sltm*, which has been described as modulator of estrogen induced transcription, and the cyclins *Ccnl1* and *Ccnl2* which are transcriptional regulators of pre-mRNA splicing.

Phenotypic information obtained from independent studies on animals need to be integrated in order to reliably compare results across different mice colonies and laboratory set-ups. The sources of phenotypic data used in the present study are metadatabases in which uniformity criteria and manual curation has been imposed on assembled records. The Mouse Tumor Biology Database (MTB) contains both spontaneous and induced tumor information for over 50 inbred strains, which is primarily extracted from the literature, from tumor pathology images submitted by investigators, and from routine animal health screenings of mouse colonies at Jackson Laboratory [9]. Then, is curated with the help of natural language processing tools to cope with increasing amounts of phenotype information in the literature [48]. Similarly, the Mouse Phenome Database (MPD) has developed standards for deposition of phenotypic data of mice including strain purity, study design, animal age and statistical power. Contributors are requested to provide complete measurement descriptions, experimental protocols as well as housing, diet and health status of animals. MPD curates data and computes summary statistics for each measurement in all strains [8].

Results presented here were obtained with a linear regression analysis. However, interplaying gene networks linked to phenotypes may not necessarily follow linear relationships with regard to transcript levels. Recently, a few reports have attempted to identify non-monotone or non-linear phenotype-transcriptome associations. Lin et al. (2008) proposed the coefficient of multiple determination (*R*^2^) of a natural cubic spline regression model [49]. In a related work, three correlation methods (Pearson, Spearman and Hoeffding’s D) were compared to analyze co-expressed genes. Hoeffding’s D dependence measure was found to be the best suited to identify nonlinear and non-monotonic associations [50]. These types of analytical approaches are needed to uncover causal phenotype-transcriptome connections that do not follow obvious linear behaviors.

Finally, since one of the strains showed a much higher OTF than the other three, we were interested in search for stronger gene links with this phenotype in the SWR strain. A t-test conducted between SWR versus the remaining 3 strains resulted in 530 statistically significant clones (see Additional file 1, figure 1). Of these, 373 clones were common with the regression test while 280 were coincident with the ANOVA test. The overlap between the 3 tests resulted in a list of 266 clones having a functional profile that resembled the terms described in Additional file 1, Table 2 for the regression results. On the other hand, 143 clones were exclusive in the t-test. Reduction of the latter subset resulted in 92 unique gene identities which were subjected to a GO analysis summarized in Additional file 1, table 3. The combined 10-terms list suggests the involvement of intracellular vesicle traffic, protein sorting and actin cytoskeleton dynamics in the observed high OTF of SWR strain. Indeed, oocyte meiotic maturation involves events related to spindle assembly. In somatic cells, chromosome segregation errors during mitosis may contribute to cancer development and progression [51]. The genes *App*, *Aplp2* and *Appbp2*, all related to the amyloid beta precursor protein, were recurrent in Additional file 1, table 3. The well known involvement of amyloid beta protein in Alzheimer’s disease pathogenesis may actually be due to a chromosomal instability process [52]. Analogous mechanisms may partly explain the high OTF observed in SWR mice.

## Conclusion

This work describes statistically significant variation in ovarian gene expression of four commonly studied mouse strains. We found that over 60% of these differences are linked to the biological variability observed in spontaneous ovarian tumor rates and reproductive parameters across strains. If NL is equivalent to multiparity, the inverse relationship detected between genes correlated to OTF and NL points to a protective effect of successive pregnancies. Post-transcriptional control of ovarian angiogenesis, folliculogenesis and oocyte maturation seems to be major contributors to this effect. Conversely, overexpression of protein folding genes might be considered as a susceptibility factor. These findings, in addition to the lack of association between OTF and LS -a measure of multiple ovulation- support repetitive menstrual cycling instead of repetitive ovulations as an important contributor to ovarian tumorigenesis. Further experimental research as well as development of bioinformatic and statistical tools to uncover complex phenotype-transcriptome associations is needed.

## Methods

### Animals, RNA extraction and cDNA labeling

Mouse strains BALB/c, C57BL/6, FVB and SWR were maintained at the Laboratory Animal Sciences Program, SAIC-NCI Frederick (Frederick, MD), under protocols of the Institutional Animal Care and Use Committee (IACUC). Adult (8-weeks old) females grown from trio mating-established colonies were euthanised on late metestrus phase by cervical dislocation after gaseous CO_2_ administration. Whole ovaries from 4-5 animals were removed from surrounding adipose tissue, thereafter pooled and immediately frozen in liquid nitrogen. Total RNA was extracted with Trizol (Invitrogen, CA) and directly labeled as Cyanine-3 or Cyanine-5 fluorescent cDNA using reverse transcription under conditions previously described [11].

### Microarray experiments

NIA-15K mouse cDNA microarrays were used. This is a curated collection consisting of 15,261 clones derived from expression libraries obtained from pre- and peri-implantation embryos, E12.5 female gonad/mesonephros and newborn ovaries [53]. Microarrays were spotted at the Laboratory of Molecular Technology, SAIC-NCI Frederick (Frederick, MD) with a BioRobotics Microgrid arrayer (Genomic Solutions, MI). Hybridization conditions and washes have been described elsewhere [11]. Samples were co-hybridized against a whole-newborn mouse total RNA as common reference sample using a replicated dye-swap design. A total of 24 microarray hybridizations were performed. TIFF images were captured with a GenePix 4000B fluorescent scanner (Molecular Devices, CA) and then saved for further analysis.

### Statistical and bioinformatic analysis

Scanned microarray images were extracted as GPR files using the GenePix 5.0 software and uploaded to the NCI’s Microarray Database (“mAdb”; http://nciarray.nci.nih.gov). Data files containing updated gene annotation were subjected to local (“print-tip”) loess normalization, scale adjustment, and filtering/imputation of missing values with the “DNMAD” and “PreProcessor” tools at the GEPAS server (http://www.gepas.org). Reproductive and spontaneous ovarian tumor records were extracted from the MPD at http://www.jax.org/phenome[8] and from the MTB at http://tumor.informatics.jax.org/mtbwi/index.do[9] databases, respectively. Tumor data corresponds to the “highest reported tumor frequency” in all literature records collected in MTB for each strain. Continuous tumor and reproductive data for the 4 mouse strains (see Table 1) were correlated to gene expression log_2_ ratios using linear regression analysis under multiple-test control *(FDR indep)* with the tool Pomelo II using 200,000 permutations (http://pomelo2.bioinfo.cnio.es/). The ANOVA test among the 4 strains and a t-test between SWR and the 3 remaining strains were also conducted with Pomelo II using 200,000 permutations. Gene functionality was primarily assessed with Gene Ontology (GO) terms using hypergeometric tests conducted with WebGestalt (http://bioinfo.vanderbilt.edu/webgestalt). Literature mining with HUGO approved gene symbols, associated aliases and keywords, was carried out in PubMed queried through GeneCards (http://www.genecards.org) and with SciMiner (http://jdrf.neurology.med.umich.edu/SciMiner/).

### Quantitative PCR confirmation of microarray results

Primer pairs design, cDNA preparation, thermocycling conditions and equipment has been previously described [11]. Quantification of mRNAs was based on C_T_ values, which is defined as the PCR cycle at which an increase in reporter fluorescence above baseline signal can be detected. Normalization was done with the 18S rRNA as reference transcript assayed under identical conditions respective to the gene of interest in both the test and the reference RNA samples. The ΔΔC_T-Sample_ value (ΔΔC_T-Sample_ = ΔΔC_T-Sample_ - ΔC_T-Reference_) was transformed by taking the result of the expression: If 2^(-ΔΔCT)^ - 1 > 0 then the result = 2^(-ΔΔCT)^ - 1 or else the result = -1 / 2^(-ΔΔCT)^ This calculation converted the linear range for down regulation from 1→0 to 0→(-∞), and up regulation from 1→(+∞) to 0→(+∞) in the log_2_ scale.

## Competing interests

The authors declare that they have no competing interests.

## Authors' contributions

UU designed the study, performed microarray experiments, data analysis, interpretation and wrote the manuscript; GAO handled clone collection and manufactured microarrays; GMZ carried out animal care and tissue harvest; JMC performed Q-PCR assays; JJS and DJM participated in study design and coordination, and critically revised the manuscript. All authors read and approved the final manuscript.

## Supplementary Material

Additional file 1 Contains: Table 1: Detailed microarray ratios and Q-PCR results of selected transcripts. Table 2: Functional analysis of genes correlated to all phenotypes. Table 3: Gene Ontology profile of genes differentially expressed between SWR and the remaining three strains. Figure 1: Summary of statistical tests among ovarian transcriptional profiles of four mouse strains. Click here for file

Additional file 2 Contains the complete, loess normalized dataset for 14,586 transcripts. Click here for file

Additional file 3 Contains results for all 145 clones correlated both to OTF and NL in mouse ovaries. Click here for file

Additional file 4 Functional profile of transcripts correlated both to OTF and NL in the mouse ovary Click here for file
