# Supplementary material for: Tumor and reproductive traits are linked by RNA metabolism genes in the mouse ovary: a transcriptome-phenotype association analysis
Source: BMC Genomics. 2010 Dec 22;11(Suppl 5):S1. doi: 10.1186/1471-2164-11-S5-S1 (PMC3045792; doi:10.1186/1471-2164-11-S5-S1)
Supplement: Additional file 1 — Contains: Table 1: Detailed microarray ratios and Q-PCR results of selected transcripts. Table 2: Functional analysis of genes correlated to all phenotypes. Table 3: Gene Ontology profile of genes differentially expressed between SWR and the remaining three strains. Figure 1: Summary of statistical tests among ovarian transcriptional profiles of four mouse strains. [file 1471-2164-11-S5-S1-S1.pdf]

Additional file 1. Table 1 – Detailed microarray ratios and Q-PCR results of selected transcripts

	Spp1			***Txnrd1***			***Anxa5***			***Fn1***			***C1s***			***Ctsl***			***Mt1***			***rRNA 18S***
	MA (d) [a]	MA (r) [a]	Q-PCR [b]	MA (d)	MA (r)	Q-PCR	MA (d)	MA (r)	Q-PCR	MA (d)	MA (r)	Q-PCR	MA (d)	MA (r)	Q-PCR	MA (d)	MA (r)	Q-PCR	MA (d)	MA (r)	Q-PCR	Q-PCR
BALBc	-5.07<br>-4.92<br>-1.31	-1.17<br>-5.20<br>-5.29	**-4.27**<br>26.72<br>26.81	0.14<br>-0.04<br>0.21	0.14<br>0.16<br>0.15	**2.85**<br>29.82<br>28.72	0.55<br>0.72<br>-0.31	1.01<br>0.65<br>0.56	**3.42**<br>20.74<br>20.65	-3.00<br>-2.29<br>-1.06	-0.96<br>-2.68<br>-2.86	**-1.13**<br>22.18<br>22.10	0.60<br>0.91<br>0.12	-0.01<br>0.63<br>0.79	**1.64**<br>23.97<br>23.97	1.34<br>1.25<br>1.05	0.52<br>1.65<br>1.48	**2.06**<br>20.54<br>20.92	-1.02<br>-0.82<br>-0.11	0.63<br>-1.56<br>-1.48	**0.01**<br>19.91<br>19.91	17.63<br>17.74
C57BL/6	-3.47<br>-4.64<br>-4.27<br>-3.09	-4.38<br>-4.21	**-4.86**<br>26.75<br>26.93	0.11<br>0.21<br>0.21<br>0.48	0.01<br>0.04	**1.02**<br>30.72<br>30.47	0.77<br>0.66<br>0.76<br>0.33	0.78<br>0.55	**1.90**<br>21.63<br>21.76	-2.57<br>-2.40<br>-3.01<br>-2.23	-2.45<br>-2.31	**-1.75**<br>22.31<br>22.20	1.74<br>1.53<br>0.46<br>1.48	1.30<br>0.97	**0.98**<br>24.01<br>22.24	1.33<br>1.17<br>1.72<br>1.91	1.41<br>1.09	**0.64**<br>21.58<br>21.70	-0.66<br>-0.74<br>-0.61<br>-0.38	-0.81<br>-0.96	**-1.25**<br>20.51<br>20.81	17.08<br>17.26
FVB	-1.12<br>-1.02	-0.78<br>-0.26<br>-1.06	**-2.48**<br>24.47<br>24.89	0.25<br>0.51	0.19<br>0.11<br>0.07	**0.84**<br>31.05<br>30.93	0.21<br>0.53	0.22<br>0.32<br>0.15	**0.98**<br>23.03<br>22.65	-3.46<br>-2.27	-0.65<br>-0.89<br>-1.50	**-0.83**<br>21.14<br>21.96	0.15<br>0.58	0.27<br>-0.41<br>0.37	**1.78**<br>23.41<br>23.68	0.70<br>0.44	0.79<br>0.43<br>0.35	**2.11**<br>20.09<br>20.69	-0.81<br>-0.53	-0.88<br>0.00<br>-0.82	**-0.11**<br>19.63<br>19.85	17.47<br>17.32
SWR	-3.10<br>-2.94<br>-2.86	-2.28<br>-2.86<br>-2.49	**-2.76**<br>23.92<br>23.93	0.33<br>0.41<br>0.33	-0.08<br>0.09<br>0.19	**0.78**<br>30.50<br>30.38	0.33<br>0.32<br>0.49	0.04<br>0.15<br>0.00	**0.71**<br>23.75<br>24.09	-2.11<br>-2.11<br>-1.72	-0.96<br>-1.21<br>-1.13	**-2.00**<br>22.04<br>22.20	1.18<br>1.26<br>0.91	0.68<br>0.31<br>0.87	**0.72**<br>23.90<br>24.08	1.52<br>1.37<br>1.52	0.79<br>0.83<br>1.02	**1.25**<br>20.45<br>20.83	-0.76<br>-0.80<br>-0.95	-0.79<br>-0.73<br>-0.92	**-1.36**<br>20.52<br>20.24	16.70<br>16.88
Ref.[c]	-	-	23.91<br>23.79	-	-	33.16<br>33.80	-	-	25.43<br>25.51	-	-	22.43<br>22.32	-	-	26.97<br>26.98	-	-	24.41<br>23.88	-	-	21.43<br>21.12	19.08<br>19.01

[a] Direct (d) and reverse (r) microarray (MA) normalized results are shown. Reverse ratios were multiplied by (-1) to facilitate comparison of replicates. Note that the C57BL/6 series consist of 4 direct and 2 reverse replicate microarray experiments, whereas the FVB series consisted of 2 direct and 3 reverse replicates.
[b] Quantitative PCR-derived ratios (bold font) were calculated from the average of duplicate Ct values shown below of each ratio, according to formulas described in Materials and Methods.
[c] A "common reference" experimental design was used. All microarray results shown were obtained with the same reference RNA sample (see Materials and Methods). The internal housekeeping gene was the 18S rRNA (leftmost column) and was assayed under identical conditions with respect to the measured transcripts.

Additional file 1. Table 2 - Functional analysis of genes correlated to all phenotypes [a]

Term [a]	*Count* [b]	*Fold Enrichment* [c]	*Adjusted p* [d]
BP00044:mRNA transcription regulation	201	1.39	2.1E-07
MF00042:Nucleic acid binding	187	1.60	6.1E-12
BP00048:mRNA splicing	136	1.35	1.7E-03
BP00040:mRNA transcription	135	1.50	9.9E-06
MF00224:KRAB box transcription factor	118	1.46	6.1E-04
BP00071:Proteolysis	103	1.36	1.8E-02
MF00213:Non-receptor serine/threonine protein kinase	97	1.48	2.0E-03
BP00031:Nucleoside, nucleotide and nucleic acid metabolism	96	2.01	3.6E-09
BP00104:G-protein mediated signaling	71	1.37	4.8E-02
BP00063:Protein modification	70	1.73	3.3E-04
BP00286:Cell structure	70	1.44	2.9E-02
MF00212:Other G-protein modulator	67	1.56	7.6E-03
BP00060:Protein metabolism and modification	63	1.48	3.1E-02
MF00262:Non-motor actin binding protein	61	1.59	8.9E-03
MF00101:Guanyl-nucleotide exchange factor	54	1.65	8.5E-03
BP00064:Protein phosphorylation	50	1.49	4.7E-02
BP00193:Developmental processes	44	1.53	4.6E-02
MF00242:RNA helicase	42	2.09	9.5E-04
BP00285:Cell structure and motility	38	1.80	1.8E-02
BP00077:Oxidative phosphorylation	35	1.65	4.6E-02
BP00224:Cell proliferation and differentiation	29	1.85	3.4E-02
MF00211:Kinase activator	28	2.39	2.2E-03
MF00033:Voltage-gated calcium channel	27	2.06	1.6E-02
MF00053:Other RNA-binding protein	26	2.60	1.7E-03
BP00179:Apoptosis	25	2.33	8.0E-03
BP00273:Chromatin packaging and remodeling	24	1.90	4.9E-02
MF00264:Microtubule family cytoskeletal protein	22	2.17	2.6E-02

MF00006:Interleukin receptor	20	2.16	4.0E-02
MF00275:Transcription cofactor	19	2.28	3.3E-02
MF00267:Membrane traffic protein	18	2.54	1.7E-02
BP00069:Protein disulfide-isomerase reaction	18	2.53	2.1E-02
BP00145:Small molecule transport	18	2.24	4.4E-02
BP00062:Protein folding	17	2.49	3.0E-02
BP00201:Skeletal development	16	2.46	3.5E-02
MF00069:Ribonucleoprotein	12	3.50	1.7E-02
BP00018:Other amino acid metabolism	10	3.56	3.6E-02
BP00284:Hematopoiesis	7	4.70	4.9E-02

[a] Performed in the DAVID Bioinformatic Resources 2008 (http://david.abcc.ncifcrf.gov/) using the PANTHER´s database categories.
[b] Note that gene identity is usually redundant between functionally related terms.
[c] Defined with a Fischer exact test ($p<0.01$) to determine random occurrence with respect to the complete mouse genome.
[d] P-value corrected with the Benjamini test for multiple test adjustment.

Additional file 1. Table 3 – Gene Ontology profile of genes differentially expressed between SWR and the remaining three strains [a]

Term [a]	***Genes*** [b]	***p*** [c]
Endomembrane system	Bscl, Ulk1, Appbp2, Chst12, Ccar1, Copz1, Nup160	8.16E-04
Establishment of protein localization	Stx18, Lman2, Appbp2, Gdi1, Copz1, Sh3glb1, Atg9a	2.64E-02
Apoptosis	App, Ubqln1, Ccar1, Cfdp1, Cd1d1, Sh3glb1	4.35E-02
Ubiquitin cycle	Ubqln1, Herc1, Rbbp6, Usp9x, Atg9a	1.45E-02
Golgi apparatus	Stx18, App, Chst12, Copz1, B4galt1	9.06E-03
Organelle envelope	Mosc2, Csde1, Ccar1, Nup160, Sh3glb1	3.00E-02
mRNA processing	App, Snrpf, Tsen34, Sf3b1	9.75E-03
Actin cytoskeleton	Actb, Csrp1, Tpm1, Cald1	1.07E-02
Extracellular matrix organization and biogenesis	App, Aplp2, B4galt1	3.01E-03
Suckling behavior	App, Aplp2	1.50E-02

[a] A t-test with FDRindep and 200,000 permutations was done with Pomelo II (see Methods). Statistically significant genes not common with the other tests (ANOVA and Regression) were subjected to a GO analysis with WebGestalt (http://bioinfo.vanderbilt.edu/webgestalt/).

[b] Note that gene lists displays some redundancy between functionally related terms.

[c] GO analysis used an hypergeometric test. GO categories were selected from a Direct Acyclic Graph with a threshold p-value ≤ 0.05.

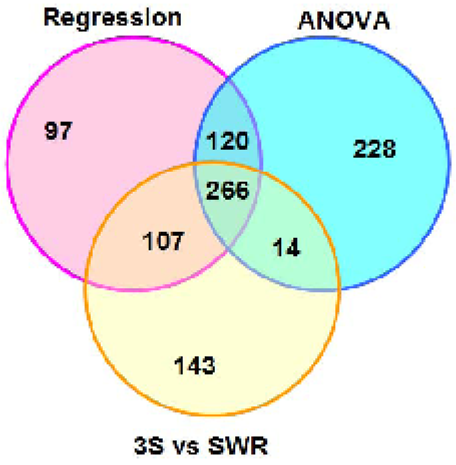

Additional file 1. Figure 1 – Summary of statistical tests among ovarian transcriptional profiles of four mouse strains

Statistically significant results of the regression analysis (590 clones), ANOVA (628 clones) and a t-test (530 clones) with FDR control. Regression and ANOVA were done between the four strains while the t-test was performed between SWR and the remaining three strains (3S vs SWR). The Venn diagram depicts coincidence and divergence between tests. The t-test results not common with the Regression and the ANOVA results were reduced to a list of 91 unique gene identities which were subjected to the functional analysis described in Supplementary Table 3.
