# Supplementary material for: Tumor and reproductive traits are linked by RNA metabolism genes in the mouse ovary: a transcriptome-phenotype association analysis
Source: BMC Genomics. 2010 Dec 22;11(Suppl 5):S1. doi: 10.1186/1471-2164-11-S5-S1 (PMC3045792; doi:10.1186/1471-2164-11-S5-S1)
Supplement: Additional file 4 — Functional profile of transcripts correlated both to OTF and NL in the mouse ovary [file 1471-2164-11-S5-S1-S4.pdf]

Additional file 4 - Functional profile of transcripts correlated both to OTF and NL in the mouse ovary [a]

OTF (-) NL (+) [a]	RB	RP	RT	ZB	UC	PF	b	Relevant biological or disease role (PMID) c
Cpsf6, cleavage and polyadenylation specific factor 6	■	■					o	Linked to tumor stage in ovarian cancer (18636107)
Ddx17, DEAD (Asp-Glu-Ala-Asp) box polypeptide 17	■						o	Transcriptional co-activator for ER alpha (11250900)
Fubp1, far upstream element (FUSE) binding protein 1	■		■				o	Regulates c-myc transcription (16628215)
Hnrpdl, heterogeneous nuclear ribonucleoprotein D-like	■		■					nrd (§)
Hnrpa2b1, heterogeneous nuclear rnp A2/B1	■	■					o	Prevents apoptosis in ovarian and other cancers (14633690)
Pabpn1, poly(A) binding protein, nuclear 1 (#)	■	■				■	m	Mutated in oculopharyngeal muscular dystrophy (17110089)
Rbm4, RNA binding motif protein 4	■	■		■			n	Decreased in the fetal brain in Down's Syndrome (12469345)
Rbm4b, RNA binding motif protein 4B	■	■		■				nrd (§)
Rbm6, RNA binding motif protein 6	■							Involved in non-small cell lung cancer (11085536)
Rbm12, RNA binding motif protein 12	■							Altered in meibomian cell carcinoma (17889501)
Rbm25, RNA binding motif protein 25	■						o	Stimulates proapoptotic *Bcl-x* splicing (18663000)
Rbm26, RNA binding motif protein 26	■			■			b	Overexpressed in T-cell lymphomas and leukemia cells (11149944)
Rbm39, RNA binding motif protein 39	■	■	■				o	Coactivates steroid hormone receptors (15694343)
Sfrs2, splicing factor, arginine/serine-rich 2	■	■					o	Expressed in human ovarian cancer (15069551)
Sfrs6, splicing factor, arginine/serine-rich 6	■	■					o	Upregulates antiangiogenic VEGF isoforms (18843117)
Sfrs7, splicing factor, arginine/serine-rich 7	■	■		■			e	Involved in GnRH pre-mRNA splicing (16249178)
Sfrs11, splicing factor, arginine/serine-rich 11		■						Modulates alternative splicing of Tau mRNA (16943417)
Sfrs12, splicing factor, arginine/serine-rich 12		■					n	Down-regulated in Alzheimer disease's brain (15456940)
Sfrs18, splicing factor, arginine/serine-rich 18		■						nrd (§)
Sltm, SAFB-like, transcription modulator	■		■					Pro-apoptotic effect and inhibition of mRNA synthesis (17630952)
Son, SON DNA binding protein	■						b	Cellular growth factor in leukemogenesis (18952841)
Srrm1, serine/arginine repetitive matrix 1	■	■						Regulates alternative splicing of the CD44 mRNA (16354706)
Tardbp, TAR DNA binding protein	■	■	■				n	Roles in cystic fibrosis and neurodegenerative disorders (17981595)
Tra2a, transformer 2 alpha homolog (*)	■	■					e	Role in estrogen mediated scavenger receptor splicing (17673517)
Ttc14, tetratricopeptide repeat domain 14	■							nrd (§)
3300001P08Rik, RIKEN cDNA 3300001P08 gene		■					o	Overexpression is associated to cisplatin resistance (10631324)
Arnt, aryl hydrocarbon receptor nuclear translocator			■				o	Binds to HIF-1alpha to recognize VEGF promoter (17208433)
Ccnl1, cyclin L1			■					Oncogene candidate in head and neck cancer (12414649)
Ccnl2, cyclin L2 (#)		■	■					Induces apoptosis in hepatocellular carcinoma cells (14684736)
Dmtf1, cyclin D binding myb-like transcription factor 1			■					Possible tumor suppressor (17237816)
Mll3, myeloid/lymphoid or mixed-lineage leukemia 3			■	■			b	Involved in leukemogenesis and developmental disorders (11718452)
Mll5, myeloid/lymphoid or mixed-lineage leukemia 5				■			b	Essential for regulation of hematopoiesis (18952892)
Mycbp2, MYC binding protein 2			■	■	■		m	Plays a key role in neuromuscular development (18255027)
Nfat5, nuclear factor of activated T-cells 5			■					Involved in hyperosmotic stress response (15965902)
Suv420h2, suppressor of variegation 4-20 homolog 2 (*)			■					Implicated in epigenetic dysregulation of breast cancer (16322686)
1810007M14, RIKEN cDNA 1810007M14 gene			■					nrd (§)
2010111I01, RIKEN cDNA 2010111I01 gene				■				Aminopeptidase involved in peptide processing (15687497)
Ece1, endothelin converting enzyme 1				■			o	Modulates prostaglandin-F2 production in pregnancy (16524686)
Luc7l2, LUC7-like 2 (S. cerevisiae)				■				nrd (§)
Zc3h7a, zinc finger CCCH type containing 7 A				■				Increased during macrophage activation (18682727)
Zc3h11a, zinc finger CCCH type containing 11A				■				nrd (§)

Gene	RB	RP	RT	ZB	UC	PF	Tissue	Reference
Zfp644, zinc finger protein 644				■				nrd ([§])
Zfp820, zinc finger protein 820				■				nrd ([§])
Zufsp, zinc finger with UFM1-specific peptidase domain				■				nrd ([§])
Otud7b, OTU domain containing 7B				■	■			Prevents interleukin-8 mediated inflammation response (18178551)
Ube2e1, ubiquitin-conjugating enzyme E2E 1					■		**m**	Upregulated in rat model of cardiac isquemia (15665047)
Usp15, ubiquitin specific peptidase 15					■			Involved in cell cycle control (18346885)
Wsb1, WD repeat and SOCS box-containing 1					■		**e**	Modulates thyroid metabolism (15965468)

OTF (+) NL (-) [a]

Gene	RB	RP	RT	ZB	UC	PF	Tissue	Reference
**BC003993**, cDNA sequence BC003993		■						nrd ([§])
Lmo1, LIM domain only 1				■			**b**	Involved in normal and pathological leukemogenesis (15198727)
Sall4, sal-like 4 (*)			■	■			**n**	Implicated in congenital and neurological disorders (16545361)
Nfia, nuclear factor I/A			■				**n**	Regulates CNS development (19058033)
Cited1, Cbp/p300-interacting transactivator			■				**o**	Regulated by FSH in human granulosa cells (16556681)
Hsp90aa1, heat shock protein 90 a, class A, member 1						■	**o**	Overexpressed in ovarian endometriosis (16815388)
Dnajb1, DnaJ (Hsp40) homolog, subfamily B, member 1						■	**o**	Involved in ovarian development in *Drosophila* (18718532)
P4hb, prolyl 4-hydroxylase, beta polypeptide (#)						■	**o**	Participates in procollagen synthesis in the ovary (17495471)

[a] The 145 clones correlated with both OTF and NL (see Table 2) were reduced to 96 unique gene identities after merging repeats and excluding unknowns, non-coding, and non-annotated clones. Based on the direction of correlation these 96 genes were further decomposed in sub-lists of 76 OTF(-) NL(+) and 20 OTF(+) NL(-) genes. This table shows the genes in enriched functional domains (56 out of the 96).

[b] Functional analysis was done with WebGestalt (http://bioinfo.vanderbilt.edu/webgestalt) using the hypergeometric test. Enriched gene ontology (GO) terms and their respective *p* values are as follows: RB= RNA binding (9.4e-16), MP= mRNA processing (1.0e-14), RT=regulation of transcription (6.6e-4), ZB=zinc ion binding (9.3e-4), UC=ubiquitin cycle (3.4e-2), PF=protein folding (1.5e-3). Tissue/organ codes are: o=ovarian; m=muscle; n=neural; b=blood; e=endocrine (non-ovarian).

[c] PubMed was queried through GeneCards using "gene symbol/alias" and "ovary/ovarian" when corresponded. The PMIDs for the most informative entries are shown.

[§] Not reported to date. Most of these cases correspond to recently identified genes for which the GO term was inferred from electronic annotation.

# At least one of the functional terms did not directly appeared in the GO database but was deducted from the literature.

* Corresponds to *Drosophila* homolog genes.
